# Safety of Laparoscopic Cholecystectomy for Acute Cholecystitis in the Elderly: A Multivariate Analysis of Risk Factors for Intra and Postoperative Complications

**DOI:** 10.3390/medicina57030230

**Published:** 2021-03-02

**Authors:** Dragos Serban, Bogdan Socea, Simona Andreea Balasescu, Cristinel Dumitru Badiu, Corneliu Tudor, Ana Maria Dascalu, Geta Vancea, Radu Iulian Spataru, Alexandru Dan Sabau, Dan Sabau, Ciprian Tanasescu

**Affiliations:** 1 4th Department of Surgery, University Emergency Hospital Bucharest, 050098 Bucharest, Romania; lulu.tudor@gmail.com; 2 Faculty of Medicine, “Carol Davila” University of Medicine and Pharmacy, 020021 Bucharest, Romania; bogdan.socea@umfcd.ro (B.S.); cristian.d.badiu@gmail.com (C.D.B.); ana.dascalu@umfcd.ro (A.M.D.); getavancea@gmail.com (G.V.); raduspataru@yahoo.com (R.I.S.); 3 Department of Surgery, “Sf. Pantelimon” Emergency Hospital, 021659 Bucharest, Romania; 4 Department of Surgery, “Bagdasar Arseni” Clinical Emergency Hospital, 041915 Bucharest, Romania; 5 Department of Pediatric Surgery, Emergency Clinic Hospital for Children “Maria S. Curie”, 41451 Bucharest, Romania; 6 3rd Department Surgery, Faculty of Medicine, “Lucian Blaga” University Sibiu, 550169 Sibiu, Romania; alexandru.sabau@ulbsibiu.ro (A.D.S.); dan.sabau@ulbsibiu.ro (D.S.); ciprian.tanasescu@ulbsibiu.ro (C.T.)

**Keywords:** acute cholecystitis, laparoscopic cholecystectomy, elderly, safety

## Abstract

*Background and Objectives:* This study investigates the impact of age upon the safety and outcomes of laparoscopic cholecystectomy performed for acute cholecystitis, by a multivariate approach. *Materials and Methods:* A 2-year retrospective study was performed on 333 patients admitted for acute cholecystitis who underwent emergency cholecystectomy. The patients included in the study group were divided into four age subgroups: A ≤49 years; B: 50–64 years; C: 65–79 years; D ≥80 years. *Results:* Surgery after 72 h from onset (*p* = 0.007), severe forms, and higher American Society of Anesthesiologists Physical Status Classification and Charlson comorbidity index scores (*p* < 0.001) are well correlated with older age. Both cardiovascular and surgical related complications were significantly higher in patients over 50 years (*p* = 0.045), which also proved to be a turning point for increasing the rate of conversion and open surgery. However, the comparative incidence did not differ significantly between patients aged from 50–64 years, 65–79 years and over 80 years (6.03%, 9.09% and 5.8%, respectively). Laparoscopic cholecystectomy (LC) was the most frequently used surgical approach in the treatment of acute cholecystitis in all age groups, with better outcomes than open cholecystectomy in terms of decreased overall and postoperative hospital stay, reduced surgery related complications, and the incidence of acute cardiovascular events in the early postoperative period (*p* < 0.001). *Conclusions:* The degree of systemic inflammation was the main factor that influenced the adverse outcome of LC in the elderly. Among comorbidities, diabetes was associated with increased surgical and systemic postoperative morbidity, while stroke and chronic renal insufficiency were correlated with a high risk of cardiovascular complications. With adequate perioperative care, the elderly has much to gain from the benefits of a minimally invasive approach, which allows a decreased rate of postoperative complications and a reduced hospital stay.

## 1. Introduction

As the world population is aging, there is an increased surgical demand for elderly people. Geriatric surgery is presently a topic of research, as many surgeons acknowledge there are specific features regarding the type of surgery, the duration and intensity of treatment and the significant complications related to the therapeutic approach at advanced ages. The term of “frailty” is often used to describe a vulnerability, a lack of resilience of the elderly to stress and increased demands upon the function of organs or systems. [1,2,3] Understanding the specific age-related challenges may help improve perioperative care by a multidisciplinary approach [3,4,5].

Acute cholecystitis is one of the most frequent conditions requiring abdominal surgery in emergencies in elderly people [6]. The current guidelines recommend surgery as soon as possible because evidenced-based clinical studies confirmed that an early treatment reduces the total hospital stay and does not increase the complication or conversion rates [7,8,9,10,11].

Laparoscopic cholecystectomy has become the “gold standard” due to its undeniable advantages in reducing pain and postoperative complications. Together with the development of anesthesia and intensive care skills and techniques, the safety limit for performing laparoscopy has also increased nowadays towards the age of 80–85 years.

In previously published studies on the results of laparoscopic cholecystectomy in the elderly, the age considered as a threshold differs: some studies consider it to be 65 years [12,13,14], 70 years [15] or 75 years [16], while several studies refer to outcomes of laparoscopic cholecystectomy in extreme ages, such as over 80 years of age [6,17,18,19,20,21]. Most studies compare the conversion rate and the incidence of postoperative complications in groups of young vs. elderly patients. There are limits in terms of reporting the results, as the effect of age is difficult to be dissociated from the presence of comorbidities, which are obviously more common with aging. Other studies [14,22,23,24] compared the complications of laparoscopic vs. classical cholecystectomy in elderly patients and found better outcomes with a minimally invasive approach.

This study aims to investigate the impact of age upon the safety and outcomes of laparoscopic cholecystectomy performed for acute cholecystitis, by a multivariate approach. The novelty factor is that age is analyzed in correlation with the anesthetic-surgical systemic risk factors and with the severity of the infectious process. The preoperative variables which correlate best with surgical decisions and postoperative outcomes were analyzed.

## 2. Materials and Methods

### 2.1. Study Design

A 2-year retrospective study was performed on the patients admitted in the 4th Department of Surgery, Emergency University Hospital Bucharest for acute cholecystitis who underwent emergency cholecystectomy, between January 2018 and December 2019. Data were collected from observation charts and postoperative notes.

The diagnosis of acute cholecystitis was assessed according to Tokyo Guidelines, based on clinical findings (Murphy sign; right upper quadrant pain, tenderness, palpable mass, fever), laboratory inflammation tests and an ultrasound exam confirming gallstones and thickness of the gallbladder wall. The inclusion criteria for the study consisted of: (I) emergency admission for acute cholecystitis followed by cholecystectomy during the same hospital admission, (II) accurate documentation of the clinical signs, paraclinical data, surgery and complications. Exclusion criteria were: (I) associated pancreatitis or any (II) malignancy.

The preoperative evaluation of the anesthetic-surgical risk was based on the American Society of Anesthesiologists Physical Status Classification (ASA PS). The severity of acute cholecystitis was evaluated according to Tokyo Guidelines criteria (TG13/TG18) (Table 1). Charlson Comorbidity Index (CCI) scores were calculated retrospectively for the patients enrolled in the study based on the comorbidities documented in the observation charts.

The management of acute cholecystitis was according to the Tokyo Guidelines 2018 flowchart [25] based on the severity of symptoms, ASA and CCI index. Emergency laparoscopic cholecystectomy was performed as soon as possible to be performed safely, within a time frame of 96 h after the admission. Broad spectrum intravenous antibiotic therapy was used in all cases. In mild cases, we used intravenous ceftriaxone (1 g/12 h), and in medium and severe cases we used a combination of ceftriaxone or piperacillin/tazobactam (4 g + 0.5 g/8 h) and metronidazole (1 g/12 h). The antibiotic therapy was initiated in emergency and continued up to 24–48 h postoperatively, in cases with a favorable outcome. In cases with pyocholecystitis, parietal micro-abscesses, or pericholecystic abscess, bile was sent for a microbiological exam, and antibiotic therapy was adjusted later in correlation with the antibiogram. Low-molecular-weight Heparin for thrombosis prophylaxis was used as a routine pre and postoperatively during the hospital stay, according to body weight and comorbidities, in doses starting from 0.4 mL/day to 1.2 mL/day.

Conversion to open surgery was used as a second option of bailout procedure, after “fundus first”, when technical difficulties were encountered and critical view of safety in the Calot triangle was not achieved. Subtotal cholecystectomy was considered a technical solution in difficult cases, and it can be performed either laparoscopically or by open surgery, depending on the surgeon’s experience and the local technical conditions. Drainage was used in all these patients.

Patients with ASA ≥ 3 and CCI ≥ 6 or sepsis underwent fluid rebalance and general supportive care before surgery.

### 2.2. Data Comparison and Statistical Analysis

The patients included in the study group were divided into 4 age-subgroups: A: ≤49 years; B: 50–64 years; C: 65–79 years; D: ≥80 years.

The main outcomes were: mortality rate and incidence of major systemic and surgery related complications. Secondly, the rate of laparoscopic cholecystectomies and the rate of conversion were analyzed comparatively in the four age-subgroups. A statistical analysis was performed to assess the association correlations between age and anesthetic-surgical risk, the severity forms of acute cholecystitis and post-operatory outcomes.

Pearson chi square, Fisher’s exact test and the Linear-by-Linear association test (Mantel-Haenszel test for trend) were used to evaluate the association between discrete variables, the ANOVA test was used for continuous variables and Fisher’s linear discriminant analysis was used for multivariate analysis. IBM SPSS Statistics 22 was applied.

In order to describe the preoperative and intraoperative patients’ characteristics which determined the applied surgical procedure (LC = Laparoscopic Cholecystectomy, Conversion or OC = Open Cholecystectomy), we have used the stepwise variant of Fisher’s linear discriminant analysis. The Canonical Discriminant Function is displayed in standardized form in order to allow the comparison of the importance of each variable. Cross-validation models were used to evaluate the statistical power of discrimination.

## 3. Results

### 3.1. Demographic Data and Preoperative Evaluation

A total of 345 patients, aged between 18 and 91 years, were admitted in emergency with the diagnostic of acute cholecystitis during January 2018 and December 2019. A total of 12 patients (3.47%) did not undergo cholecystectomy during the same hospital admission and were excluded from the statistical analysis. In one case (0.28%), a man aged 87, with severe cardiac insufficiency and sepsis (ASA IV), emergency cholecystostomy was performed. Drainage of the bilious-purulent content of the gallbladder allowed recovery in a case in which general anesthesia was considered not appropriate due to high risk of death. Conservative management was used in 11 cases (3.18%). Four cases refused surgery (aged between 42 and 83 years), while in seven cases cholecystectomy was postponed by the surgeon for various reasons (Table 2).

**Figure 1:**
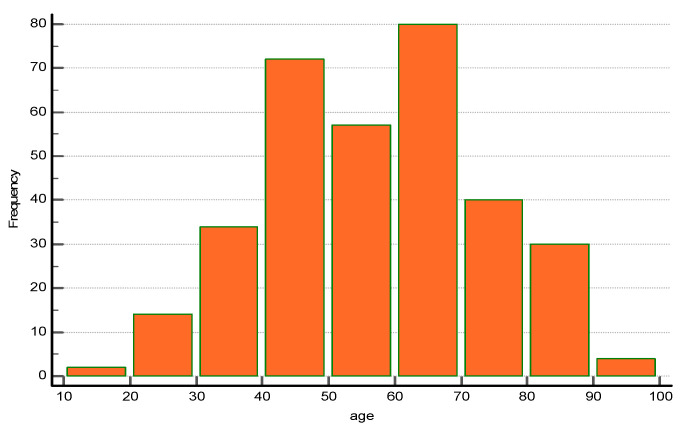
Age distribution of patients with emergency cholecystectomy for acute cholecystitis in the study group (*n* = 333).

A total of 333 patients (96.54%) underwent emergency cholecystectomy and were further included in the statistical analysis. The distribution of patients follows a multiple peak pattern, suggesting the overlay of multiple populations (Figure 1).

There were no statistically significant differences in terms of gender distribution in the four subgroups (Table 3). Presentation at more than 72 h after onset was considered a sign of severity of the level of local inflammation according to the Tokyo Guidelines. In the study group, there was a upward trend correlated with age and surgery after 72 h from onset, confirmed by the Linear-by-Linear association test (*p* = 0.007).

**Figure 2:**
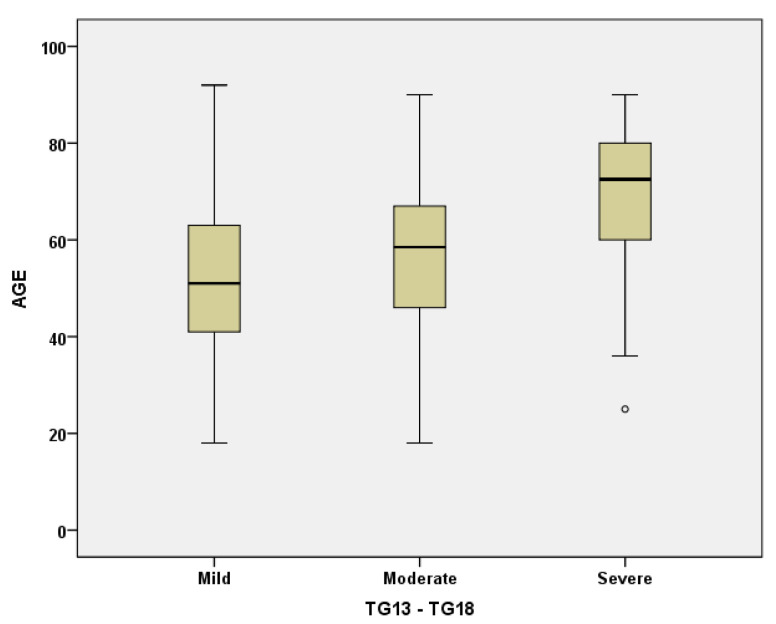
Boxplot representation of age distribution by Tokyo Guidelines TG13/TG18 Classification.

The moderate forms (TG 13/18) were the most frequent in all age groups. However, the statistical analysis showed a tendency for the mild forms to decrease with age, with a corresponding increase in the severe forms with organ/system decompensation (Figure 2), with statistically significant differences being observed between group A on the one hand and groups C and D on the other hand (*p* < 0.001). The same differences were observed for the leukocytes > 18,000/mmc and fibrinogen > 400 mg/mL.

The age of 65 represents a statistically significant demarcation limit in terms of associated comorbidities and anesthetic-surgical risk. CCI correlates well with age (Spearman rho 0.462, *p* < 0.001). In groups C and D compared to groups A and B, there were significantly fewer patients with ASA PS risk I and significantly more patients with ASA PS ≥ 3, with the increase in the ASA score with age being confirmed by the Linear-by-Linear association test (*p* < 0.001).

The incidence of signs of acute cardiac insufficiency at admission significantly increased with age, from 2.5% in group A to 44.1% in group D. Similar correlations were found with creatinine levels > 1.2 mg/mL, an expression of a pre-existing age-related limitation of renal function, with decompensation in the context of systemic inflammation and sepsis. There were only five cases with INR > 2. It correlated with chronic anticoagulant therapy for cardiovascular associated comorbidities.

### 3.2. Surgical Approach and Postoperative Outcomes

As general management, the laparoscopic approach was the first choice for all patients of all ages. Open cholecystectomy was performed only when laparoscopy was not considered safe due to comorbidities or local conditions.

We noted a statistically significant difference between the age distribution for LC compared to OC and conversion: the mean age for LC is 55, while the mean age for OC and conversion is 68 (*p* < 0.001 for ANOVA test). However laparoscopic cholecystectomy was the most frequent procedure in all subgroups, with superior outcomes when compared to open surgery and conversion in terms of hospital stay and surgical and cardiovascular complications (*p* < 0.001).

Furthermore, the linear-by-linear association shows an increase in the conversion rate with age (*p* < 0.001). The frequencies of the conversion rate and the classic surgical approach were significantly higher in patients aged over 50.

Conversion to open was a surgical decision due to elective (lack of advancing in dissection and specimen removal, lack of critical view of safety—20 cases) or emergent causes (incontrollable hemorrhages—four cases; main bile duct lesion—one case, cholecystic-duodenal fistula—one case). In the present study, we found no statistically significant differences between conversion and open cholecystectomy in terms of mortality, morbidity and hospital stay. In the case of intraoperative main bile duct lesion, the conversion was imposed by the difficult dissection due to chronic inflammation of the cystic pedicle. The lesion was situated in the proximity of the cystic duct and was classified as minor according to the Mc Mahon Classification (<25% of main bile duct diameter) and was repaired by a T tube insertion. Large papillosphincterotomy was performed by endoscopic retrograde cholangio-pancreatography (ERCP) in the early postoperative period (3 days later) to allow faster recovery.

The classic approach of first intention was used in a total of 12 cases (one in group A, four in group B, five in group C, two in group D). The causes for open surgery were: increased local inflammation (gangrenous gallbladder, biliary peritonitis) in eight cases, the association of the main biliary duct lithiasis with mechanical jaundice ± angiocholitis (two cases) and a history of previous surgical interventions in the upper abdominal region (two cases) (Table 4).

The cases in which drainage of the subhepatic space was considered necessary were those cases with severe local inflammation, increased intraoperative bleeding or suspected lesion of the bile duct. The fact that the drain was used more often in the elderly is well correlated with the increased incidence of the moderate and severe forms with advanced age. Drainage was used in all cases with open surgery and conversion to open.

The postoperative outcome was favorable in most cases for all age subgroups. No patients required re-surgery in the following 30 days. Surgical related complications were managed conservatory: hemorrhages (seven cases), bile leakage (nine cases), one septic intraperitoneal collection and one main bile duct lesion, classified as minor according to the Mc Mahon Classification solved by ERCP stenting. The procedure consisted of papillosphincterotomy, and a plastic material 7F stent of 10 cm length was introduced in the main bile duct to allow healing. The stent was removed after 3 months, with a favorable outcome. Surgical site infections were less common in laparoscopic cholecystectomy vs. open cholecystectomy and conversion (Table 5), and increased with age.

The rate of surgery related complications was significantly higher in patients over 50 years old (*p* = 0.045), which also proved to be a turning point for an increasing rate of conversion and open surgery. However, the comparative incidence did not differ significantly between patients aged from 50–64 years, 65–79 years and over 80 years. (6.3%, 9.09% and 5.8%, respectively).

The Fisher’s linear discriminant analysis was performed to identify the risk factors significantly related to surgical complications. The highest correlation was found with systemic comorbidities: diabetes (r = 0.813) and chronic bronchopneumopathy (r = 0.502) and CCI (r = 0.381, but with no significant increase in discrimination power). Among the local factors, the severity of inflammation and the presence of gangrenous cholecystitis had the most significant predictive power (r = 0.288), followed by fibrinogen (r = 0.348), and TG13/TG18 severity forms (r = 0.218).

Severe cardiovascular complications encountered in the study group were: acute myocardial infarction (nine cases), stroke (seven cases) and malign arterial hypertension (two cases). In total, three out of four causes of death were cardiovascular acute events. Only one patient died of sepsis: a diabetic patient aged 57 with a severe form of acute cholecystitis. The incidence of severe cardiovascular postoperative complications increased with age (ANOVA test for linearity: *p* < 0.001; Mantel-Haenszel test for trend: *p* < 0.001). There were no statistically significant differences between the incidence of cardiovascular complications in groups B, C and D (*p* = 0.344).

### 3.3. Multivariate Analysis of Risk Factors for Open Surgery and Conversion

In order to describe the preoperative and intraoperatory patients’ characteristics which determined the applied surgical procedure (LC = Laparoscopic Cholecystectomy, Conversion or OC = Open Cholecystectomy), we have used the stepwise variant of Fisher’s linear discriminant analysis. The discrimination between the classes is based on the two Canonical Discriminant Function described in Table 6. The Canonical Discriminant Function is displayed in standardized form in order to allow the comparison of the importance of each variable.

The variables significantly correlated with Standardized Canonical Discriminant Function F1 are gangrenous cholecystitis (r = 0.807), leukocytes (r = 0.650), fibrinogen, and severity form classified by TG 13/18. The variables significantly correlated with Standardized Canonical Discriminant Function F2 are total bilirubin (r = 0.637), CCI (r = 0.531) and high aspartate transaminase (AST) and alanine transaminase (ALT) (r = 0.351), previous history of stroke (r = 0.296), diabetes (r = 0.223) and cardiovascular disease (r = 0.236). The parameters not included in the definition of F1 and F2 are clinically significant, but they do not add a supplementary increase in the discrimination power.

F1 could be labeled as the score of inflammatory risk (higher values of leukocytes, the presence of severe inflammation and higher age imply high values if F1), and F2 could be labeled as the score of comorbidities (CCI and associated pathologies). Main bile duct complications, such as lithiasis, angiocholitis, and Mirizzi Syndrome (characterized by increased bilirubin), but also increased inflammation with a secondary increase in bilirubin, are also associated with Function 2.

**Figure 3:**
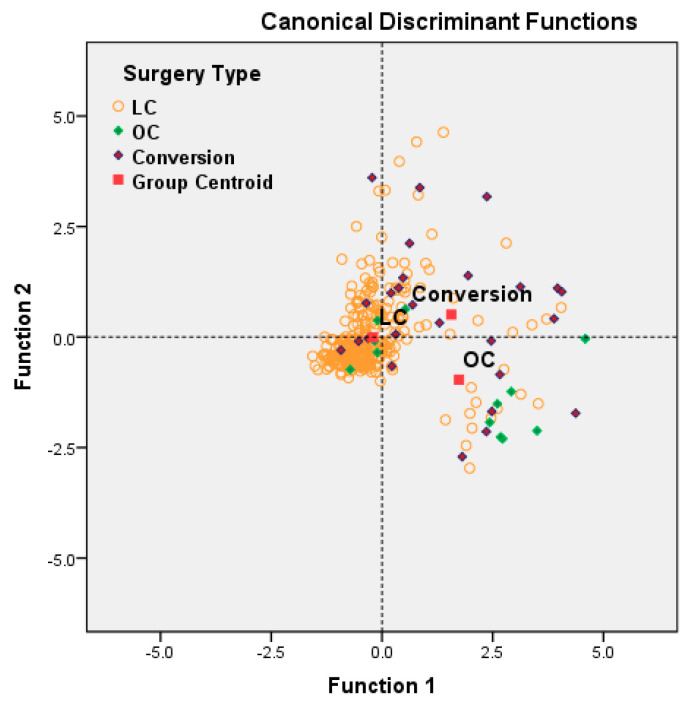
Patient representation (*n* = 333) in the space (F1, F2) of Unstandardized Canonical Discriminant Functions between (LC, OC, Conversion). (Specification: functions at Group Centroids are: (−0.203, −0.004) for LC (laparoscopic cholecystectomy); (1.739, −0.964) for OC (open cholecystectomy) and (1.564, 0.508) for Conversion). Wilks’ lambda computed for the two canonical functions are significant (test of the two functions: chi-square (df = 10) 111.08, *p* < 0.001; test of second function: chi-square (df = 4) = 17.05, *p* = 0.002). Cross-validation of the model: 81.4% of cross-validated cases are correctly classified. The relative dispersion of patients with conversion to open surgery indicates that other factors, such as surgical experience or particular intraoperative findings, may be involved.

Figure 3 suggests the following simple interpretation: small and moderate values of F1 and F2 (near zero) generally characterize the laparoscopic approach; positive values of F1 (severe inflammation and sepsis) and negative values of F2 generally characterize the open approach; and positive values of F1 and F2 (association with severe inflammation and comorbidities/main bile duct complications) generally characterize conversion.

### 3.4. Multivariate Analysis of Risk Factors for Adverse Outcome in the Eldery

The incidence of acute cardiovascular events in the early postoperative period increases statistically significantly in patients with ASA ≥ 3, and that of deaths in ASA ≥ 4 (*p* = 0.001). When the correlations between the severe forms of acute cholecystitis and the occurrence of complications were analyzed, statistical analysis showed that severe forms with organ/system dysfunction correlated with the incidence of severe complications and deaths, for all age groups.

Regarding the type of operation, the incidence of cardiovascular complications is significantly higher in the case of the open approach and conversion in comparison with laparoscopic cholecystectomy. However, conversion and open surgery were chosen in severe forms, with necrotic gallbladder, pericholecystic plastron or biliary peritonitis. Multivariate analysis of preoperative and intraoperative risk factors shows that the incidence of severe cardiovascular complications and deaths correlates best with the severity of the septic process and inflammation (gangrenous cholecystitis, fibrinogen > 400 mg/dL and Grade III cholecystitis according to TG13/18 severity forms), and among comorbidities, with a previous history of stroke, chronic renal failure and diabetes (Table 7).

## 4. Discussion

Increased technical experience with laparoscopic cholecystectomy favorably affected outcomes over time [26]. Together with the important achievements in intensive care, more patients, initially considered at risk, can benefit from the important advantages of minimally invasive surgery. The present contraindications for laparoscopic cholecystectomy are few, and they may be classified as absolute (uncorrected coagulopathy, high anesthetic and surgical risk, gallbladder carcinoma) or relative. The latter includes either general conditions (end-stage liver disease) or local findings (previous surgery in the upper abdominal region, calcified gallbladder, cholecysto-enteric fistula, Mirizzi’s syndrome) [27]. Age and severe inflammatory forms, such as gangrenous and emphysematous cholecystitis, are no longer considered unsuitable for laparoscopy [28]. In the present study, we analyzed the factors that influence the surgical decision the most. We found that severe local inflammation as well as a high CCI and high values of total bilirubin could favor open surgery or conversion. Other unquantifiable factors such as local anatomy, tissue friability, or surgeon’s experience may play a significant role in the decision to convert to open.

Hyperbilirubinemia significantly increases the likelihood of finding common duct stones in patients with acute cholecystitis, but it also occurs in patients with acute cholecystitis without common duct stones. In these cases, the increase in value is mild and it returns to normal values quickly after resolving the septic process. The significance of bilirubin in acute cholecystitis and other intraperitoneal infections was also investigated by other authors [29,30,31,32,33,34,35,36]. Hyperbilirubinemia in acute abdominal infections is caused either by the excessive production of bilirubin or by altered clearance. Both mechanisms lead to bilirubin accumulation and play a role in the hyperbilirubinemia observed in patients with appendicular perforation. Patients in severe sepsis express proinflammatory cytokines, with cholestasis triggered by nitric oxide, by blocking bilirubin conjugation and elimination at the hepatocellular and intraductal level [32]. Common pathogens of the biliary and digestive wall, such as Escherichia coli and Bacteroides fragilis, were supposed to interfere with hepatocyte microcirculation, inducing sinusoidal lesions [35]. In addition, Escherichia coli infection has been shown to induce hemolysis of normal erythrocytes. This results in increased bilirubin loading in infected individuals, a process that promotes hyperbilirubinemia [34,35,36].

There are concerns about using the laparoscopic approach in patients with respiratory and cardiovascular comorbidities due to the metabolic effects of the induced pneumoperitoneum. This loss of reserve capacity is the single most important factor that decreases the elderly patient’s ability to tolerate operations. The proper management of fluid and electrolyte replacement, respiratory management to prevent atelectasis and pneumonia, and monitoring for possible cardiac complications are necessary to minimize the risk of systemic complications in the perioperative period [2,3,37,38].

### 4.1. Comparative Characterization of the Age-Related Subgroups

The comparative statistical analysis of the four subgroups defined according to age showed that each of them behaves differently and presents specific challenges and outcomes.

Group A, of young patients (<50 years), is a group without significant comorbidities and without significant anesthetic-surgical risk, which generally presents with mild and moderate forms resolvable in a proportion of 97.5% laparoscopically, with a short postoperative stay and without significant complications. In the presence of a septic factor, they can still develop severe cardiovascular acute events and even death. Fluid and electrolyte rebalancing and supportive care were important as an adjuvant to combat septic shock.

Group B (50–64 years) did not differ statistically significantly from group A in terms of anesthetic-surgical risk and CCI score. The severity of the forms of acute cholecystitis was not significantly increased, but there were patients with longer biliary distress with local fibro-inflammatory remodeling, which explains the intraoperative technical difficulties, with an increased conversion rate (7.2% vs. 1.6% in group A) and the classic approach by open cholecystectomy (3.6% vs. 0.8%). During the early postoperative period, these patients were at risk of major cardiovascular complications, especially when diabetes or chronic renal disease are associated.

Group C (65–79 years) was characterized by a statistically significant increase in both the anesthetic-surgical risk (ASA-PS and CCI) compared to group A, but also a significant increase in severe cases according to TG13/TG18 criteria (12.1% vs. 2.5%, *p* = 0.001). Recall that severe forms of acute cholecystitis mean the association of significant local and general inflammation with systemic or organ dysfunction. This result therefore correlated with significant increases in biological markers of inflammation (leukocytosis, fibrinogen) compared to group A. Additionally, the presence of increased CCI and associated comorbidities, especially cardiovascular disease and diabetes, explained the evolution of cholecystitis from moderate to severe, with functional decompensation. In the therapeutic management of these patients, careful preoperative rebalancing was particularly important to prevent major systemic complications and reduce perioperative mortality.

Group D (>80 years) presented the same clinical-therapeutic challenges as group C, but the differences from group A were more marked: late presentation, higher frequency of severe forms of TG 13/18, anesthetic-surgical risk increased by the presence of comorbidities, having as outcomes an increased rate of conversions and major postoperative systemic complications. Thus, the conversion rate increased from 1.6% in group A to 17.6% in group D, and open surgery from 0.8% to 5.9%. However, there were no statistically significant differences in terms of preoperative evaluation and surgery approach and postoperative outcomes between group C (65–79 years) and group D (≥80 years).

Consequently, patients over 50 years of age in the presence of cardiovascular comorbidities or diabetes should be closely monitored in the postoperative period to avoid cardiovascular ischemic incidents and cardiovascular decompensation.

The utility of drain insertion in laparoscopic cholecystectomy is still a subject of debate. In a recent systematic review, Cirrochi et al. [39] found that the incidences of wound infection and abdominal collections are significantly higher in the drain group vs. the no-drain group, while the postoperative recovery and hospital days are shorter in cases without drain. In our clinic, drain insertion was not a routine procedure after laparoscopic cholecystectomy. However, it is still used in cases with severe inflammation, difficult dissection or bleeding in order to prevent intra-abdominal collections in the early postoperative period. An increased incidence of drain insertion with age was well correlated with the severity of acute cholecystitis in the elderly. This could also be an explanation for the increased incidence of postoperative septic complications, such as wound infection and intra-abdominal collections, described by other authors [40,41,42].

### 4.2. Safety of Laparoscopic Cholecystectomy in the Elderly

Although laparoscopic cholecystectomy is currently considered to be a routine abdominal procedure with minor risks, a deep understanding of the physiological reserve of elderly patients is also mandatory in surgery, as it can be used to assess the vulnerability of patients with frailty syndrome to complications [1,2,3,20].

Acute cholecystitis has clinical particularities in aged patients: statistically significant increases in severe forms, as well as the presence of associated comorbidities, with an increased rate of conversion and a higher percentage of postoperative complications. These findings were also encountered in other studies [19,20,21,24,43,44,45]. In a crossectional analysis on cholecystectomy in the elderly, Kuy et al. found that older people have more complex forms of disease and that a longer time from admission to surgery is a predictor for poor outcome [43].

In a meta-analysis on 99 studies between 1995 and 2018, Kamarajah et al. [44] found a tenfold increase in mortality in patients aged over 80. One of the major drawbacks they remarked on in their research was that the studies evaluated did not take into account the associated comorbidities and their impact on the final outcomes. In a meta-analysis on 11 studies published between 1993 and 2011, on 101,559 patients aged 65 or older (48,195 treated laparoscopically and 53,364 by open cholecystectomy), Antoniou et al. found that mortality was 1.0% for the laparoscopic approach and 4.4% for the open approach [24].

In the present study, there were 100 patients aged over 65, and 77% of them successfully underwent laparoscopic cholecystectomy, with 0% mortality. In the 23 cases in which laparoscopy could not be performed (direct open surgery and conversion groups), there was only one death (4.34%). In our study, despite an increased conversion and complications rate, there were no deaths in group D (aged over 80). There were no significant differences regarding cardiovascular complications between the four age-groups. Similar findings are also encountered by Shin et al. [38]. With the pre-operative optimization of comorbidities and medications and addressing frailty in a multi-disciplinary team, an experienced surgical staff with good technical equipment are effective in improving postoperative outcomes [16,38]. Moreover, the multivariate analysis showed that severe inflammation (gangrenous cholecystitis) and comorbidities such as diabetes, previous stroke and chronic renal and pulmonary disease, but not age itself, are risk factors for postoperative morbidity. This finding is also communicated by Kim et al. [46,47]. Moreover, Agrusa et al. recommended elective laparoscopic surgery in elderly people with symptomatic gallstone disease before the development of acute cholecystitis and related complications [48].

When comparing open to laparoscopic surgery, most of the studies found better outcomes in terms of mortality and morbidity associated with laparoscopic procedures [15,24,49], while a limited number of studies founded similar results for both methods [22]. These findings confirmed that laparoscopic cholecystectomy is a safe procedure and should be used in the elderly. On the other hand, a proper comparison cannot be performed between the open and laparoscopic approach due to the fact that the open approach does not represent a first line option in our surgical department, regardless of the patient’s age. Open surgery (and conversion) was used only in cases in which laparoscopic surgery could not be performed. The severity of the inflammatory process and sepsis might also be associated with increased mortality in the open surgery group.

Laparoscopy is associated with a limited response in serum Il-6 and no change in gut mucosa Il-6 [50]. There is strong evidence that laparoscopy provides a decreased inflammatory response at the peritoneal and intestinal level, with a faster intestinal transit recovery. The reduced inflammatory systemic response associated with laparoscopic surgery may also be important, especially in the elderly, in preventing pulmonary related complications [50].

## 5. Conclusions

The present study showed that laparoscopic cholecystectomy is the most used surgical approach in the treatment of acute cholecystitis in all age groups, with better outcomes than open cholecystectomy in terms of decreased overall and postoperative hospital stay, reduced surgical related complications and a reduced incidence of acute cardiovascular events in the early postoperative period. On the other hand, patients with a higher ASA grade and severe forms of TG 13/18 were more likely to undergo open surgery.

Laparoscopic cholecystectomy can be safely performed in elderly and extremely elderly people, but the risk of severe postoperative cardiovascular complications is slightly higher. Careful perioperative care of the vascular, hemodynamic and respiratory status should be provided in order to prevent these adverse events in the elderly. The degree of systemic inflammation and sepsis was one of the main factors that influenced the adverse outcome of LC in the elderly. Among comorbidities, diabetes was associated with both increased surgical related and cardiovascular postoperative morbidity, while a previous history of stroke and chronic renal insufficiency are correlated with a high risk of cardiovascular complications. CCI, ASA PS and the incidence of severe forms increase with age, also leading to slightly more complications. However, age alone should not be the contraindication for laparoscopic cholecystectomy. With adequate perioperative care, the elderly have much to gain from the benefits of a minimally invasive approach, which allows a decreased rate of postoperative complications and a reduced hospital stay.

## Figures and Tables

**Table 1:** Tokyo Guidelines (TG13/TG18) severity risk scale [9,25].

Grade III (severe) acute cholecystitis	Acute cholecystitis with organ/system (renal, cardiovascular, hepatic, respiratory, neurologic, hematologic) dysfunction
Grade II (moderate) acute cholecystitis	Acute cholecystitis associated with: WBC * > 18,000/mmc Palpable tender mass in the right upper abdominal quadrant Marked local inflammation Onset > 72 h
Grade I (mild) acute cholecystitis	Acute cholecystitis which does not meet criteria for grade II or III

* WBC—white blood cells.

**Table 2:** Demographic and clinical data of non-operated patients.

No.	Age	TG 13/18 Severity Form	Reason for Postponed Surgery	Returned for Elective Surgery during the Study Period
1	37	mild	Refused surgery	no
2	39	mild	Associated giant right renal cyst; deferred to urology after conservative management	yes, 4 months later
3	53	moderate	Neglected arterial hypertension *	yes, after one month
4	53	mild	Refused surgery	no
5	57	moderate	Morbid obesity (BMI ** 43)	no
6	61	mild	Ultrasound (US) and Computed tomography (CT) exam raised suspicion of gallbladder carcinoma	yes, for further evaluation and elective oncological surgery
7	64	mild	Morbid obesity (BMI 41)	no
8	69	mild	Refused surgery	yes, 6 months later
9	72	mild	US and CT exam raised suspicion of colon cancer	yes, for further evaluation and elective oncological surgery
10	82	moderate	Increased anesthetic risk due to severe cardiac insufficiency	no
11	86	mild	Refused surgery	no

* hypertension—Blood pressure (BP) of 22 mmHg at admission. As the patient responded to medical therapy for acute cholecystitis, he was referred to a cardiologist and asked to return for elective surgery, under adequate medication. ** BMI – body mass index.

**Table 3:** Demographic and preoperative data in the 4 age-subgroups.

Group	A	B	C	D	Total	*p* Value
Age (years)	≤49	50–64	65–79	≥80	18–91	
Number	122	111	66	34	333	
Onset > 72 h	59.8%	69.4%	75.8%	79.4%	68.2%	*p* = 0.007 ^(1)^
Female (%)	29.5%	29.7%	36.4%	26.5%	30.6%	*p* = 0.716 ^(2)^
Severity forms TG13/TG 18						
Mild	36.10%	27%	22.70%	11.80%	27.90%	*p* < 0.001 ^(1)^
Moderate	61.50%	68.50%	65.20%	70.60%	65.50%	
Severe	2.50%	4.50%	12.10%	17.60%	6.60%	
Leukocytes ≥ 18,000/mmc	2.5%	7.2%	12.1%	14.7%	7.2%	*p* = 0.025 ^(2)^
Fibrinogen > 400 mg/dL	34.4%	48.6%	60.6%	67.6%	47.7%	*p* = 0.007 ^(1)^
Creatinine > 1.2 mg/dL	19%	19.8%	37.9%	50%	26.2%	*p* < 0.001 ^(1)^
Aspartate transaminase (AST), Alanine transaminase (ALT) > 40 UI/L	33.6%	38.7%	28.8%	47.1%	35.7%	*p* = 0.268 ^(2)^
INR(international normalized ratio) > 2	0	2.7%	0	5.9%	1.5%	*p* = 0.039 ^(2)^
Bilirubin > 1.2 mg/dL	11.4%	9.9%	24.2%	29%	15.31%	*p* = 0.045 ^(2)^
Sign of acute cardiac insufficiency at admission ***	2.5%	9.9%	21.2%	44.1%	12.9%	*p* < 0.001 ^(1)^
Neurologic decompensation at admission	0	0	0.015%	0.029%	0.006%	N/A
ASA PS risk						
I	33.60%	18%	6.10%	0	19.50%	*p* < 0.001 ^(1)^
II	54.10%	57.70%	53%	44.10%	54.10%	
III	12.30%	20.70%	37.90%	44.10%	23.40%	
IV	0	2.70%	3%	8.80%	2.40%	
V	0	0.90%	0	2.90%	0.60%	
CCI						
0	88.40%	60.30%	28.70%	20.50%	58.80%	*p* < 0.001 ^(1)^
1	5.70%	14.40%	27.20%	26.50%	15.30%	
2	1.60%	14.10%	21.20%	20.60%	8.40%	
3	5.70%	11.70%	12.10%	8.80%	9.30%	
4	1.60%	3.60%	4.50%	14.70%	5.10%	
5	0	1.80%	3%	0	1.20%	
≥6	0.80%	1.80%	0	8.80%	1.80%	

Footnote: ^(1)^ Test of Linear-by-Linear Association; ^(2)^ Fisher’s exact test; ASA PS: American Society of Anesthesiologists Physical Status Classification; TG13/18: Tokyo Guidelines classification risk; CCI: Charlson Comorbidity Index. *** described according to Common Guide of diagnostic and treatment of Acute Cardiac Insufficiency of European Society of Intensive Therapy and European Society of Cardiology: (i) Aggravated preexisting cardiac insufficiency (edema of the lower limbs, congestion); (ii) Hypertensive Cardiac insufficiency (high BP, tachycardia, signs of vasoconstriction); (iii) Pulmonary acute edema: acute respiratory disfunction, with tachypnea and orthopnea, SaO_2_ < 90% before oxygen administration; (iv) Acute coronary syndrome; (v) Cardiogenic shock: hypotension requiring vasopressor medication, signs of organ hypoperfusion, with oliguria.

**Table 4:** Surgical approach and postoperative outcomes in the 4 subgroups.

Group	A (<50 Years) *n* = 122	B (50–64 Years) *n* = 101	C (65–79 Years) *n* = 66	D (>80 Years) *n* = 34	Total *n* = 333	*p* Value
Type of surgery						
LC	119 (97.5%)	99 (89.2%)	51 (77.3%)	26 (76.5%)	295(88.6%)	*p* < 0.001 ^(1)^
Conversion	2 (1.6%)	8 (7.2%)	10 (15.2%)	6 (17.6%)	26 (7.8%)	
OC	1 (0.8%)	4 (3.6%)	5 (7.6%)	2 (5.9%)	12 (3.6%)	
Drainage in LC	8 (6.72%)	9 (9.09%)	12 (21.05%)	9 (34.6%)	36 (12.2%)	*p* < 0.001 ^(1)^
Hospital days (mean ± SD *)						
Total	4.65 ± 3.03	6.35 ± 3.03	6.53 ± 3.9	7.4 ± 4.4	6 ± 3.35	*p* < 0.001 ^(2)^
LC	4.58 ± 2.21	5.38 ± 2.7	5.83 ± 3.47	5.66 ± 2.53	5.51 ± 2.9	
Conversion	6.8 ± 2.77	9.2 ± 3.52	11.42 ± 4.5	12.2 ± 5.01	9.92 ± 4.15	
OC	9	9 ± 5.56	7.25 ± 3.26	10.8 ± 3.6	9.15+/4.15	
Postoperative hospital days (mean ± SD)						
Total	3.46 ± 2.27	3.75 ± 3.43	4.22 ± 3.53	5.35 ± 4.1	3.63 ± 2.8	*p* < 0.001 ^(2)^
LC	2.49 ± 1.46	2.68 ± 1.7	2.75 ± 1.81	3.83 ± 1.91	3.12 ± 2.22	
Conversion	5.2 ± 2.77	6.72 ± 2.63	9.14 ± 4.45	10 ± 5.33	7.73 ± 3.9	
OC	6	8 ± 3.6	5.75 ± 3.77	10.8 ± 3.6	6.92 ± 2.92	

^(1)^ Fisher’s exact test; ^(2)^ ANOVA Linearity test; LC: laparoscopic cholecystectomy; OC: open cholecystectomy; Drain insertion was not a routine practice in our clinic for laparoscopic cholecystectomy; * SD—standard deviation.

**Table 5:** Postoperative complications according to Clavier-Dindo Classification.

Clavier-Dindo Classification	A (<50 Years) *n* = 122	B (50–64 Years) *n* = 101	C (65–79 Years) *n* = 66	D (>80 Years) *n* = 34	Total *n* = 333	*p* Value
I (surgical site infections)						
Total	1 (0.81%)	3 (3.03%)	3 (4.53%)	2 (5.71%)	5 (3.05%)	*p* < 0.001 ^(1)^
LC	1 (0.8%)	2 (2%)	2 (3.92%)	1 (3.85%)	6 (2%)	
conversion	0	1 (12.5%)	1 (10%)	0	2(7.6%)	
OC	0	0	0	1 (50%)	1 (8.3%)	
II (surgical related complications, treated pharmacological)						
Total	2(1.6%)	6 (%)	5 (%)	2 (5.8%)	16 (%)	*p* < 042 ^(1)^
LC	1 (0.84%)	2 (%)	2 (3.9%)	1 (3.8%)	7 (%)	
conversion	0	3 (37.5%)	2 (%)	1 (16.6%)	6 (%)	
OC	1 (100%)	1 (25%)	1 (20%)	0	3 (25%)	
III (surgical related complications requiring endoscopic/surgical/Rx approach)						*p* = 1 ^(1)^
Total	0	1 (0.9%)	1(1.5%)	0	2 (0.6%)	
LC		1 (1%)	0		1 (0.33%)	
conversion		0	1 (10%)		1 (3.84%)	
OC		0	0		0	
IV (requiring intensive care)						*p* < 0.344 ^(1)^ *p* < 0.001 ^(2)^
Total	3 (2.4%)	7 (6.3%)	5 (7.57%)	3 (8.8%)	18 (5.4%)	
LC	1 (0.8%)	4 (4.04%)	3 (5.8%)	2 (7.6%)	10 (3.36%)	
conversion	1 (50%)	0	1 (10%)	1 (16.6%)	3 (11.5%)	
OC	1 (100%)	3 (75%)	2 (40%)	0	6 (50%)	
V (Deceased)						
Total	1 (0.81%)	2 (1.8%)	1 (1.51%)	0	4 (1.2%)	*p* = 1 ^(1)^
LC	0	1 (1.01%)	0	0	0.33%	
conversion	1 (50%)	1 (12.5%)	0	0	8.33%	
OC	0	0	1 (20%)	0	7.69%	

^(1)^ Fisher’s exact test; ^(2)^ ANOVA Linearity test; LC: laparoscopic cholecystectomy.

**Table 6:** Standardized Canonical Discriminant Functions for (LC, OC, Conversion).

Standardized Canonical Discriminant Function Coefficients		
	Standardized Function	
	F1	F2
Age	0.300	−0.151
Bilirubin	0.127	0.711
Leukocytes	0.426	0.173
Gangrenous cholecystitis	0.637	−0.523
CCI	0.094	0.661

**Table 7:** The Fisher’s linear discriminant analysis for cardiovascular severe complications and mortality.

Standardized Canonical Discriminant Function Coefficients	
	Function
	1
Gangrenous cholecystitis	0.211
Stroke	0.785
Diabetes	0.249
Chronic renal insufficiency	0.264
Fibrinogen > 400 mg/dL	0.348
Grade III Cholecystitis (TG13/TG18 Severity forms)	0.163

## Data Availability

Not applicable.
